# Adherence to guidelines for testing and treatment of children with pharyngitis: a retrospective study

**DOI:** 10.1186/s12887-018-0988-z

**Published:** 2018-02-09

**Authors:** Thea Brennan-Krohn, Al Ozonoff, Thomas J. Sandora

**Affiliations:** 1 Division of Infectious Diseases, Department of Medicine, Boston Children’s Hospital, Harvard Medical School, 300 Longwood Avenue, Boston, MA 02115 USA; 2 Center for Applied Pediatric Quality Analytics, Boston Children’s Hospital, Boston, MA USA

**Keywords:** *Streptococcus pyogenes*, Pharyngitis, Antibacterial agents, Antibiotic use, Antimicrobial stewardship

## Abstract

**Background:**

Group A streptococcus (GAS) is the most common bacterial etiology of pharyngitis but is difficult to distinguish clinically from viral pharyngitis. There are benefits to early antibacterial treatment of GAS pharyngitis, but administering antibiotics to children with viral pharyngitis is ineffective and costly. We evaluated adherence to guidelines that were developed to help clinicians distinguish between viral and GAS pharyngitis and guide management.

**Methods:**

Retrospective cohort study of patients ages 3–18 who had a rapid streptococcal test and/or throat culture performed in an outpatient setting. We collected data on documentation of components of the McIsaac score and classified tests as indicated if the score was ≥2. Based on McIsaac score and GAS test results, we determined whether each antibiotic course prescribed was indicated according to the Infectious Diseases Society of America guideline.

**Results:**

Among 291 eligible children, 87 (30%) had all five components of the McIsaac score documented. There was sufficient data to classify the score as either < 2 or ≥2 in 234 (80%); among these, 96% of tests were indicated. Twenty-nine patients (10%) were prescribed antibiotics. Eight (28%) of these prescriptions were not indicated according to guidelines.

**Conclusions:**

The majority of GAS tests in children with pharyngitis are indicated, although providers do not regularly document all elements of a validated pharyngitis scoring tool. Over one quarter of children prescribed antibiotics for pharyngitis did not require antibiotics according to guidelines. There remains a role for targeted antimicrobial stewardship education regarding pharyngitis management in pediatric outpatient settings.

## Background

Group A streptococcus (GAS) is the most common bacterial etiology of pharyngitis, accounting for approximately one quarter of cases of pharyngitis that bring children to medical attention [1, 2]. Acute rheumatic fever (ARF) and suppurative complications of GAS can be prevented by early antibacterial treatment [3]; indeed, prevention of ARF is one of the primary goals of antibiotic treatment of GAS pharyngitis [4]. However, GAS pharyngitis is difficult to distinguish clinically from viral pharyngitis. Treating children who have viral pharyngitis with antimicrobials is ineffective, generates unnecessary costs, exposes them to antibiotic side effects without benefit, and contributes to the growing problem of antimicrobial resistance [5]. To minimize prescription of antimicrobials for viral pharyngitis, clinical scoring systems have been developed to predict the likelihood of GAS infection [6–8]. Among these is the McIsaac score, which was developed and validated in both children and adults [9, 10]. Such scores have low positive predictive values, but help identify patients at low risk of GAS, in whom testing is not only unnecessary but may lead to identification of chronic GAS carriers experiencing viral pharyngitis [11].

The Infectious Diseases Society of America (IDSA) guideline on diagnosis and management of GAS pharyngitis recommends that patients whose clinical presentation is consistent with GAS pharyngitis be tested with a streptococcal rapid antigen detection test (RADT) or throat culture; treatment is indicated if either is positive. Testing is not recommended for patients whose presentation is most consistent with a viral etiology [12]. The American Academy of Pediatrics (AAP) has made similar recommendations [13]. Nonetheless, studies evaluating the management of pharyngitis among pediatric providers have identified high rates of antibiotic prescribing [14], even for patients with negative GAS tests [15]. To our knowledge, no study in a pediatric population has yet evaluated adherence to IDSA guidelines using individual patients’ clinical data and test results.

## Methods

### Study design and criteria

We performed a retrospective cohort study of patients seen at Boston Children’s Hospital (BCH) who had a RADT and/or streptococcal throat culture performed in an outpatient setting (hospital-affiliated primary care or urgent care clinic or emergency department [ED]) from August 1, 2011 to July 31, 2012. The first 50 patients meeting inclusion criteria in each month of the study period were evaluated. Patients were excluded if they were < 3 or > 18 years of age, were diagnosed with another bacterial infection during the visit, had a medical condition likely to cause deviation from typical pharyngitis management (e.g. neutropenia, airway compromise), were already taking antibiotics, or had been treated for GAS pharyngitis within the previous 30 days. Patients were also excluded if there was no visit documentation associated with the test; these included patients seen at local pediatric offices that use the BCH laboratory but have separate medical record systems. Information was obtained through review of chart documentation, including notes, vital signs, lab results, and prescriptions. The study was approved by the Committee on Clinical Investigation at BCH.

### Data collection

Data abstracted from medical records included information about the visit (month, day of week, time, and location), the patient (age, gender, antibiotic allergies, comorbid illnesses, reason(s) for visit, maximum reported temperature in the previous 48 h, history of cough, concurrent antibiotics, and treatment for GAS pharyngitis within the previous 30 days), physical exam findings (temperature, tonsillar exudate and/or enlargement, cervical lymphadenopathy), test results (RADT, throat culture), and management (including antibiotic prescription details). Statements regarding treatment and management decisions were also recorded.

### Calculation of McIsaac score

We used the clinical score developed by McIsaac [9] to characterize each patient’s likelihood of GAS infection. In this score, one point each is assigned for temperature > 38 °C, absence of cough, tender anterior cervical adenopathy, tonsillar swelling or exudate, and age 3–14 years. We considered patients to have had a fever if they reported fever at home or had a temperature of > 38 °C at the visit. We assigned a point for tonsillar swelling when providers documented “enlarged tonsils”, “hypertrophied tonsils” or an equivalent phrase, or recorded tonsillar size of 3+ or greater. All patients with documented cervical adenopathy were assigned one point for this element, regardless of whether tenderness or anterior location was specified. Patients were then assigned to one of three categories: McIsaac score < 2 (very low risk of GAS infection), ≥2, or indeterminate. If not all relevant data were documented, we categorized the score as indeterminate unless the category (≥2 or < 2) could be determined from known elements (e.g. if 4 of 5 items were documented as negative, the score must be either 0 or 1 and therefore was classified in the < 2 category).

### Outcomes and statistical analysis

The primary outcome was the proportion of tests indicated by the IDSA guideline. We classified a test as indicated if the McIsaac score was ≥2, not indicated if the score was < 2, and indeterminate if it could not be assigned to one of these categories, as described above.

For patients prescribed antibiotics, we classified the prescription as indicated if the McIsaac score was ≥2 and the RADT and/or GAS throat culture was positive, not indicated if the tests were negative or if the McIsaac score was < 2 (regardless of RADT and GAS results), and indeterminate if the McIsaac score was indeterminate and the RADT and/or GAS culture was positive. Patients prescribed antibiotics empirically following a negative RADT were not considered to have received antibiotics if they were instructed to stop antibiotics when the culture result returned negative. We classified each antibiotic as a recommended or non-recommended agent according to the IDSA guideline [12]. For patients without a penicillin allergy, recommended antibiotics are penicillin V, amoxicillin, or IM benzathine penicillin G; for patients with a penicillin allergy, recommended antibiotics are cephalexin, cefadroxil, clindamycin, azithromycin, or clarithromycin. We calculated the proportion of prescribed antibiotics that were recommended agents. Statistical analysis was performed using R software v3.1 (R Foundation, Vienna, Austria).

## Results

### Patient characteristics and test results

**Fig. 1:**
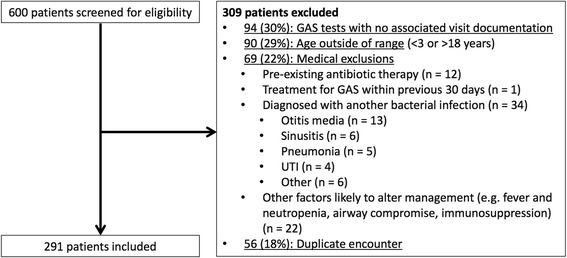
Cohort assembly

Of 600 charts reviewed, 291 patients met inclusion criteria (Fig. 1). One hundred forty-six patients (50%) were female, and the median age was 8 years (interquartile range, 5–13). One hundred forty-one patients (48%) were seen in the ED and the remainder in outpatient clinics. Twenty-one patients (7%) had positive GAS tests, of which 5 (24%) were RADTs; the other 16 (76%) had negative RADTs but positive cultures. A history of sore throat, throat pain and/or difficulty swallowing was documented for 195/291 (67%) of patients; there was no significant difference in positive test rates between patients with and without a documented complaint of sore throat [16/195 (8.2%) vs. 5/96 (5.2%); *P* = 0.35].

### Indicated and non-indicated tests

Two hundred twenty-four of 291 GAS tests (77%, 95% CI: 71–82%) were indicated, 10 (3%, 95% CI: 2–6%) were not indicated, and 57 (20%, 95% CI: 15–25%) were indeterminate. Excluding indeterminate tests, 224/234 tests (96%, 95% CI: 92–98%) were indicated. The distribution of McIsaac scores is shown in Table 1. Among patients with positive GAS results, tests were indicated in 19/21 and indeterminate in 2/21. Throat culture was performed in all patients who had a negative RADT.

**Table 1:** Distribution of McIsaac Scores

McIsaac score or score range	Number (percentage) of patients (*n* = 291)
0	0 (0)
1	7 (2.4)
2	24 (8.2)
3	24 (8.2)
4	24 (8.2)
5	8 (2.7)
0–1	3 (1.0)
0–2	4 (1.4)
0–3	3 (1.0)
0–4	0 (0)
0–5	0 (0)
1–2	25 (8.6)
1–3	16 (5.5)
1–4	7 (2.4)
1–5	2 (0.7)
2–3	46 (15.8)
2–4	24 (8.2)
2–5	2 (0.7)
3–4	46 (15.8)
3–5	14 (4.8)
4–5	12 (4.1)

### Documentation of components of the McIsaac score

All patients’ ages were available in the electronic medical record. For 2/291 patients (0.7%), this was the only component of the score documented. For 12/291 patients (4%), 2 components were documented, for 58/291 patients (20%) 3 components were documented, and for 132/291 (45%) 4 components were documented. All 5 components were documented for 87/291 patients (30%). Only one chart documented use of a clinical scoring system (in this case, the Centor score).

**Fig. 2:**
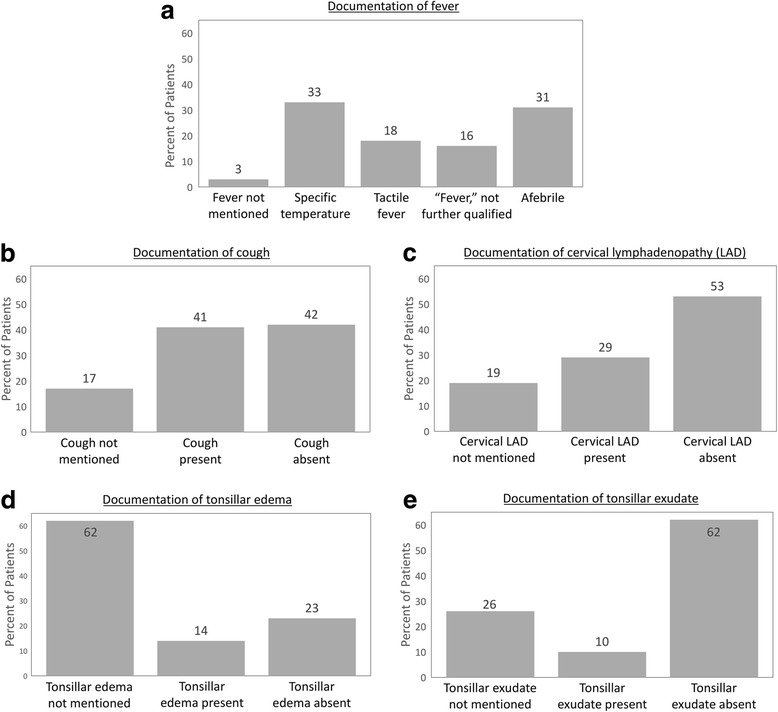
Documentation of McIsaac score components. (**a**): Fever, (**b**): Cough, (**c**): Cervical lymphadenopathy (LAD), (**d**): Tonsillar edema, (**e**): Tonsillar exudate. For each score component, the percentage of patients in whom the finding was documented as being present or absent or was not mentioned is presented. In the case of fever (panel (a)), documentation is classified according to the description of the fever

Documentation by score component is shown in Fig. 2. Temperature was recorded at 283/291 patient visits (97%). Among 84 patients noted to have cervical lymphadenopathy, presence or absence of tenderness was documented in 23 cases (27%) and the location (anterior vs. posterior) was documented in 38 (45%). Only 6 patients were specifically noted to have cervical lymphadenopathy that was anterior and tender.

### Antibiotic prescriptions

Twenty-nine of 291 patients (10%) received antibiotics, including all patients with a positive GAS test. In 27/29 cases (93%) there was sufficient documentation to categorize the test as indicated or non-indicated; in all of these cases it was indicated. Nineteen of 29 antibiotic prescriptions (66%, 95% CI: 46–81%) were indicated according to guidelines, while 8/29 (28%, 95% CI: 13–47%) were not indicated. In all cases where antibiotics were not indicated, testing was appropriate but RADT and culture results were negative. Further characterization of these patients is presented in Table 2. Of note, in the case of one patient who presented with fever and trismus and was treated with ampicillin-sulbactam upon hospitalization, antibiotic therapy may have been initiated because of concern for a peritonsillar abscess and thus could be considered appropriate for a separate indication. In one case antibiotics were prescribed in the setting of a negative RADT because of high clinical suspicion, but the family was contacted and told to stop the antibiotics when the culture returned negative. As defined above, this patient was not considered to have received antibiotics. Two of 29 prescriptions could not be classified as indicated or non-indicated because the McIsaac score was indeterminate, although in both cases the tests were positive. There were no cases of patients not receiving antibiotics when guidelines suggested they should have.

**Table 2:** Characteristics of patients who were prescribed antibiotics but should not have been according to guidelines

Age range (years)	Site	Reason(s) for visit	McIsaac score^a^	Antibiotic prescribed	Notes, including quotations from provider documentation
3–5	ED	Abdominal pain, fever, congestion	3–4	Amoxicillin	Provider mentions negative RADT and pending culture. No comment on negative culture result in chart.
3–5	ED	Rash, throat pain, rhinorrhea	2–4	Amoxicillin	“Given that patient is otherwise classic for a scarlet fever rash, will treat with amoxicillin…”
6–8	Clinic	Sore throat, fever, rhinorrhea, cough	3–4	Amoxicillin	Diagnosis in note is “viral infection”. Antibiotic was prescribed by a different provider than the one who wrote the note.
6–8	Clinic	Sore throat, fever	3–4	Amoxicillin	“Will treat… in view of impressive exam.” Upon receipt of negative throat culture result: “Throat [culture] neg[ative]. Will leave on [antibiotics] for probable tonsillitis.”
12–14	Clinic	Headache, vomiting, sore throat	3–4	Amoxicillin	“Could be viral given negative rapid strep, but symptoms are classic, so will treat presumptively…”
12–14	ED	Sore throat, fever, voice change, trismus, snoring	5	Ampicillin-sulbactam, amoxicillin-clavulanate	Diagnosed with tonsillitis, admitted to hospital. Heterophile antibody test negative. Recently treated with clindamycin for GAS-negative tonsillitis.
12–14	ED	Throat pain, ear pain, fever, rhinorrhea, epigastric pain	3	Clindamycin	Heterophile antibody test positive.
12–14	ED	Throat pain, fever, ear pain	4–5	Amoxicillin	“Rapid strep negative though Centor score would suggest high probability… Will treat empirically for strep pharyngitis.”

All patients had negative RADT and throat culture

^a^A range is provided for the McIsaac score in cases where there was insufficient clinical information in the chart to determine the exact score

### Antibiotic prescriptions: Recommended and non-recommended agents

Among patients prescribed antibiotics, 26/29 (90%, 95% CI: 72–97%) received recommended antibiotics and 3/29 (10%, 95% CI: 3–28%) received non-recommended antibiotics. Recommended antibiotics included penicillin V (1), amoxicillin (22), and azithromycin (1, in a patient allergic to penicillin and cephalexin). Non-recommended antibiotics included a second-generation cephalosporin (1), clindamycin (1), and ampicillin-sulbactam during inpatient hospitalization followed by amoxicillin-clavulanate upon hospital discharge (1).

## Discussion

Treating children with antibiotics when not indicated generates unnecessary healthcare costs and exposes patients to the risks of antibiotic treatment without associated benefit, while contributing to increasing antimicrobial resistance [5, 16, 17]. A recent AAP report on the use of antibiotics for upper respiratory tract infections, including pharyngitis, emphasized the importance of judicious prescribing in order to slow the rise of resistance [18]. Pharyngitis is an important target for antimicrobial stewardship efforts because of the large number of patients affected and because cases caused by GAS are difficult to distinguish clinically from those with a viral etiology [19]; such clinical uncertainty has been implicated in unnecessary antibiotic prescribing [20, 21].

Prior studies have evaluated adherence to guidelines for management of pharyngitis among pediatric care providers. Kronman et al. found that 57% of children received antibiotics during office visits for pharyngitis, significantly higher than the expected rate of GAS infection [2]. However, this study did not evaluate patients’ histories or GAS test results, so it was not possible to determine how many patients were prescribed antibiotics in the absence of GAS infection or to characterize patients’ clinical presentations. In an analysis of pediatric outpatient visits for pharyngitis, Benin et al. found that a GAS test was ordered for 78% of patients and that the presence of pharyngeal exudate increased the likelihood of testing; 36% of patients prescribed antibiotics had a negative GAS test [15]. However, this study did not include data on other clinical predictors of GAS infection to assess whether testing was indicated.

Our study contributes to the current literature by characterizing documentation of signs and symptoms of children presenting with pharyngitis, allowing us to determine the proportion of GAS tests and antibiotic prescriptions that were indicated based on the patient’s clinical history and microbiological results. A 2006 study by Linder et al. showed that in two-thirds of cases clinicians did not follow any published set of guidelines in managing adults with sore throat [22]. Antibiotics were prescribed for 47% of patients, when fewer than 20% of patients would have received an antibiotic had any published guideline been followed. Both non-indicated testing and prescribing contributed to the high rate of antibiotic prescriptions, and 19% of patients were prescribed a non-recommended antibiotic. To our knowledge, no such study has previously been performed in the pediatric setting.

In our study, 28% of antibiotic prescriptions for pharyngitis were not indicated. Extrapolated to the estimated 6.65 million annual antibiotic prescriptions for pharyngitis in children aged 3–17 [23], this suggests approximately 1.86 million (95% CI 0.865–3.125 million) excess antibiotic prescriptions yearly. While this is an approximation, it is clear that unnecessary antibiotic use for pharyngitis in pediatric outpatient settings remains an important area of focus on a national scale.

GAS testing was indicated in the majority of patients in our study, including those inappropriately prescribed an antibiotic, suggesting that unnecessary testing does not drive excessive prescribing, but instead that clinicians may prescribe antibiotics despite negative testing when they have a strong clinical impression that a patient has a GAS infection. In some cases such deviations from guidelines may be appropriate, as there are rare false negative results even with the combination of RADT and throat culture. Throat culture has been described as having a sensitivity of 90–95% based on evaluations of replicate cultures and comparison to antibody levels [24], but this may be lower if the throat swab is not collected using optimal technique [12]. (It is worth noting that for patients aged 3–14, who receive a point for age, only one additional criterion is required to reach a McIsaac score of 2). Thus, education regarding the importance of proper sample acquisition and the high sensitivity of a combination of RADT and throat culture performed on such a sample would likely be an important aspect of prescriber education. It is also notable that ARF is very uncommon in most developed countries, with an estimated annual incidence of 1 case per 100,000 children [25]; it is less common in teenagers [26]. Clinicians who are inclined to prescribe antibiotics for presumed GAS infection despite a negative test because they fear missing the opportunity to prevent a case of ARF may feel more comfortable accepting a negative result and forgoing treatment following education on the rarity of this condition.

Although our study is not directly comparable with the Linder study [22], it appears that the gap between ideal and actual management of pharyngitis may be smaller in pediatric patients than adults. Possible explanations include the higher frequency of GAS infection in children [27], leading to a greater familiarity with the diagnosis, or a higher level of concern about antibiotic side effects in children. Furthermore, there has been an overall decline in rates of antibiotic prescriptions for children, especially for respiratory tract infections, over the past two decades [28–30].

Our study has several limitations. Because we included only patients tested for GAS, we did not capture those treated empirically without testing. However, given the wide availability of RADT and throat culture at the sites evaluated, it seems unlikely that many patients would have been treated without testing. We may have inaccurately classified some tests as appropriate by assigning a point for cervical lymphadenopathy not specifically described as anterior and tender. However, reclassifying the data using this stricter definition did not result in substantive changes (data not shown). Assigning a point for fever to children who were afebrile in the office but reported fever at home may have inaccurately increased some scores, but only including children who were febrile in the office would likely have been inaccurately restrictive. We excluded children under 3 because the presentation of GAS infection in this age group is variable, and appropriate management is less clearly defined [12]. Our study was performed at an academic medical center and may not reflect practice in other settings. Finally, because the number of patients not managed according to guidelines was small, we were unable to assess factors contributing to non-indicated antibiotic prescriptions, such as age and location of care.

There were some unexpected findings in our study. The proportion of positive GAS tests (7%) was lower than the typical 20–30% seen in children with sore throat [1, 2], but we do not expect the overall rate of positive results to have altered clinical management of individual patients. While the typical sensitivity of an RADT is approximately 75–85% [31], only 5 of the 21 positive GAS tests in our sample, or 24%, were positive by RADT; the reason is not clear, although it may simply have been a chance result due to the small number of positive tests.

## Conclusions

In conclusion, we found that 28% of antibiotic prescriptions for pediatric patients evaluated for pharyngitis were not recommended by guidelines, primarily due to the prescription of antibiotics in spite of negative GAS test results. Given the frequency of pharyngitis in children, further targeted antimicrobial stewardship education for providers should be emphasized to reduce unnecessary antibiotic use. Studies assessing why adherence to guidelines for the management of pharyngitis in children appears to be greater than in adults may help elucidate which aspects of antimicrobial stewardship efforts have been most successful.

## Abbreviations

AAP: American Academy of Pediatrics
CI: Confidence interval
ED: Emergency department
GAS: Group A streptococcus
IDSA: Infectious Diseases Society of America
RADT: Rapid antigen detection test
